# Verification of Numerical Models of High Thin-Walled Cold-Formed Steel Purlins

**DOI:** 10.3390/ma17174392

**Published:** 2024-09-05

**Authors:** Přemysl Pařenica, Martin Krejsa, Jiří Brožovský, Petr Lehner

**Affiliations:** Department of Structural Mechanics, Faculty of Civil Engineering, VSB-Technical University of Ostrava, Ludvika Podeste 1875/17, 708 00 Ostrava, Czech Republicjiri.brozovsky@vsb.cz (J.B.)

**Keywords:** numerical analysis, roof purlins, thin-walled cold-formed steel, high Z cross-section

## Abstract

High thin-walled cold-formed steel purlins of the Z cross section are important elements of large-span steel structures in the construction industry. The present numerical study uses the finite element method to analyse the 300 mm and 350 mm high Z cross sections in-depth. The prepared numerical models are verified and validated at several levels with experiments that have been previously published. Significant agreement between the numerical models and the experimental results regarding Mises stress, proportional strain, failure mode, and force-deformation diagram have been obtained. With the verification, the presented procedure and partial findings can be applied to other similar problems. The results can be used to help research and corporate groups optimise the structural design of cold-formed thin-walled steel structures.

## 1. Introduction

The application of thin-walled (TW) cross-sections made from cold-formed steel (CFS) in the construction industry is continuously expanding [1,2]. The advantages, considering the modernisation of production and material savings [3], contrast with the disadvantages, namely the high sensitivity to imperfections [4] and stability problems [5,6,7]. The application of TW-CFS in the construction industry is extensive, including structural load-bearing elements of roofs and entire buildings, or secondary spreading elements [8,9,10]. A large field of application is in large spans of lightweight steel halls, where the advantage of low material quantity and high load-bearing capacity is fully realized due to suitable forming. The advantages mentioned above are in line with the goal of carbon neutrality and reducing the negative impact of humans on the environment. In fact, reducing the amount of building material and auto-amplification can help to achieve these goals [11].

Although many so-called standard-sized elements are used in the building industry [12], the aim is to expand the possibilities of TW-CFS and apply them to other buildings of larger or atypical dimensions—for example, the tall (300 mm or more) Z-shaped cross-sections, which can be ideal as purlins for large-span buildings [13]. Existing systems are mostly based on cross-sections up to 250 mm in height; larger profiles are used less frequently, but more importantly, they present several problems that need to be addressed.

Similarly, systems for connecting TW sections to other structural members and reinforcement methods at the stress concentration point should be considered. In fact, there are approaches suitable for lower cross-sections but not suitable for higher cross-sections. The current standard EN 1993-1-3 “Design of steel structures—Part 1–3: General rules—Supplementary rules for cold-formed members and sheeting” [14] covers information on most of the usable cross-sections in construction, but they have some limitations, mainly in the direction of connection and structural detail. In practice, structural connections are designed mainly according to technical data sheets based on extensive experimental programmes [10].

There are several approaches to solving the stability of thin-walled cross-sections using numerical methods. In general, these are the finite element method (FEM) [15], or the finite strength method (FSM). The difference between these methods is in the definition of finite elements. In the FEM, the entire structure is divided into discrete elements. On the contrary, the FSM is a simplified version in which the structure is divided into strips along the length of the element, resulting in a simpler and faster calculation. The direct strength method (DSM) [16] has been developed as an alternative to FSM. The DSM method calculates the effective stress derived from the critical yield strength, in contrast to the original FSM, where the effective width of the elements is determined by an iterative calculation procedure. The DSM method has been validated by extensive research [4,16,17]. There are several studies focused on both physical experiments and numerical modelling. For example, a research group compared experimentally and by numerical analysis two basic types of solutions for the super-support region of the Z profile roof trusses [18]. This research shows that the overlap solution is more load-bearing and achieves higher stiffness. Another conclusion of the research resulting from the presented experimentally verified data is the finding that the distribution of bending moments using a sleeve does not correspond to a continuous beam, and therefore such a design would be on the unsafe side. In another piece of research [19], the authors performed a detailed numerical analysis of the connection of purlins, focusing on the stiffness of the Z-connection of purlins with overlap. The numerical models showed sufficient stiffness of the overlap of the Z truss pairs that are commonly used in European markets. These are semi-rigid connections that have sufficient rotational capacity and can be treated as continuous beams in the global numerical model.

Other studies provide information on the analysis of the effect of longitudinal reinforcement in Z profiles [20]. In the research, the authors performed both physical experiments and detailed numerical models. As a result, they evaluated the effect of longitudinal stiffeners on the load capacity and concluded that, after confronting it with the DSM method, it is sufficiently accurate for estimating the shear and bending capacity of trusses in a particular configuration. Finally, the authors evaluated the interaction diagrams, concluding that for Z trusses it is appropriate to replace the circular interaction diagram with a trilinear one, which gives fewer conservative results and is therefore more efficient from an optimization point of view. Today, it is possible to use numerical analytical tools, such as the FEM, which allows the effective and efficient solution of problems of parts or whole building structures from TW-CFS [19,21,22]. However, in most cases, it is necessary to verify numerical models with experiments. In the ideal scenario, a combination of an extensive experimental program and an in-depth numerical analysis considering all necessary aspects are conducted [23].

An experimental program, including mechanical tests of high purlins (heights of 300 mm and 350 mm), has been previously presented in several publications [24,25]. Based on these data, a parametric study considering different approaches to FEM modelling was carried out [26]. The results of the study served as a basis usable for a detailed verification of the FEM models at the level of the experimental program. To the best of the authors’ knowledge, extensive numerical studies involving the behavior of high TW-CFR purlins applicable to large structures have not been performed. Therefore, procedures for model preparation, optimization for experimental measurements, and the resulting comparison with experimental data regarding the force-deformation diagram, failure mode, von-Mises stress, and relative strain are presented here.

## 2. Experimental Program

**Figure 1:**
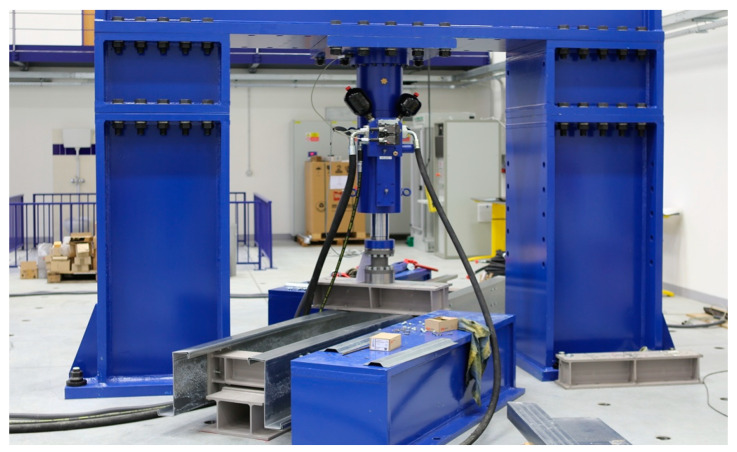
Photos of the test setup—the placement of the upside-down purlins and support matches the needs of the experiment.

The numerical analysis is based on data from previously published research [24,25] conducted by our research team (see Figure 1). The research aimed to analyse experimentally and numerically the behaviour of tall TW-CFRs at the point of connection to primary support structures. Previous published articles also included an evaluation of the applicability of standard procedures and other recommendations.

In earlier research, dimensions, material properties and general assumptions for the analysed assembly were defined [24]. Numerical models were conceived at a scale of 1:1 to best capture the behaviour of the experiments. A total of 60 combinations containing two different spans of supports (3 m and 5.1 m), two Z cross-section heights (300 mm and 350 mm), two support connection options (with and without clip), two variations of material thickness of the cross-sections (1.89 mm and 2.85 mm) and four variations of support width (180 mm, 200 mm, 250 mm and 300 mm) were tested. A note should be added here that the declared thickness of the steel plates was 1.95 mm and 2.95 mm, but during testing, the actual thickness was 1.89 mm and 2.85 mm. Therefore, these values were included in the numerical model. These combinations were based on a parametric numerical analysis study that took into account the different behaviours of the different experimental variations. The number of numerical models and the selected variants are discussed below. The experimental part of the program has been described in detail in a previous study [24], but for a complete context, the most relevant information and data are presented in this paper. Connections without and with clip are defined in detail and evaluated in the literature [21]. The research also included material testing of the steel, which determined that the 1.89 mm-thick material has a yield strength of 457.1 MPa and an ultimate strength of 538.0 MPa. For the 2.85 mm-thick material, the yield strength was 425.2 MPa and the ultimate strength was 501.8 MPa.

## 3. Numerical Analysis Assumptions

**Figure 2:**
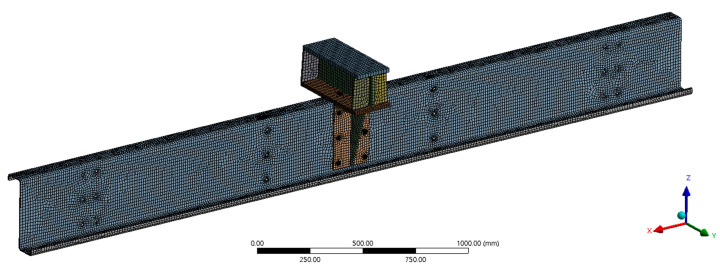
Numerical model purlins with support element and clip—finite element mesh display.

As noted above, the present parametric study is based on the assumptions and preliminary results from the initial analysis of approaches to FEM model development and evaluation presented earlier [26]. In the present paper, FEM is applied using the ANSYS 2023 R2 software [27]. Combining different approaches, such as the application of different finite element types, different material models, or different joint modelling approaches [26], led to the development of a robust numerical approach that could be used to simulate all experimental results obtained for similar purlin tests. The need to prepare all models with the following principles emerged as crucial. All models developed were performed as complex spatial models and included geometric, structural, and material nonlinearities. Nonlinear contacts were set at the points of contact between the model parts to account for friction between surfaces. As the aim was to create as accurate a model of the tested assembly as possible, it was necessary to create models of the joints that were as representative as possible of the actual behaviour of the joints in the structure. The numerical model included one purlin, a support element and alternatively a clip, or the model was without a clip (see Figure 2).

The cross-sections were modelled as shell elements—SHELL181 [27]. The joints (bolts) were modelled using beam element (BEAM188) [27], and prestressing was applied using PREST179 [27]. The connection of purlins to other structures beyond the support element was solved by remote displacement [27]. This simulates the fit of the beam as a sliding joint. In addition, it should be noted that the stabilization properties of another part of the structure are taken into account in the model.

**Figure 3:**
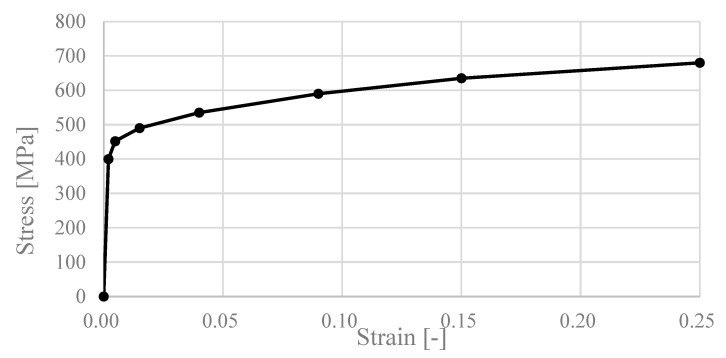
Stress–strain diagram.

The computational process regarding the loading of the structural model was divided into three parts (Load Step—LS). In LS1, the prestressing was introduced into the bolts; in LS2, the deformation load corresponding to the transverse behavioural region of the whole experimental assembly was introduced; and in LS3, the plastic behaviour, crippling and maximum load of the assembly were affected. All three LS were divided into sub-steps that provided sufficient space for convergence of the computational model results. The solution of the system of equations within the FEM was based on the Newton-Raphson method. Large deformation effects were considered during all computations. A typical model contained approximately 60,000 nodes and 40,000 elements, which led to approximately 300,000 equations. The definition of the material was based on the parameters in Table 1 and Figure 3.

## 4. Input Parameters for Numerical Models

Based on the 60 experimental tests performed, 20 numerical simulations cases were selected and prepared. These are divided into two groups: (A) with reinforcement clip, (B) without reinforcement clip. The parameters of all models are shown in Table 2. Two methods were used to label each model. The first labelling (in Table 2, column 1) was chronological according to the preparation of each model. The second method of labelling (in Table 2, column 7) was based on the editable variables of the model. For example, the model labelled as Z300_1.89_200_3.0_A has a purlin with a section height of 300; a material thickness of 1.89 mm; a support width of 200 mm; and a supports span of 3.0 m. the model includes a reinforcing clip (A). The two labelling options have the goal of better describing the results in a graphical representation.

## 5. Results of Parametric Numerical Study

The results of the numerical simulation are represented by a force-deformation diagram embedded in the graph, where it is compared with the same diagram obtained from the experiments. Furthermore, for each model, the graphical outputs from the numerical models include the deformed shape at the end of the simulation. These are compared with photographs of the actual failure modes after the load test of the given experimental setup. The graphical outputs are complemented by a von Mises stress plot and plastic ratio fields on the deformed structure. The colour scale is set at 0.2% plastic strain and 5% plastic strain limits according to EN 1993-1-5 Annex C [28]. For von Mises stresses, the colour scale for values above the average yield stress (440 MPa) is set as grey.

### 5.1. Result for Model 1A and 1B

**Figure 4:**
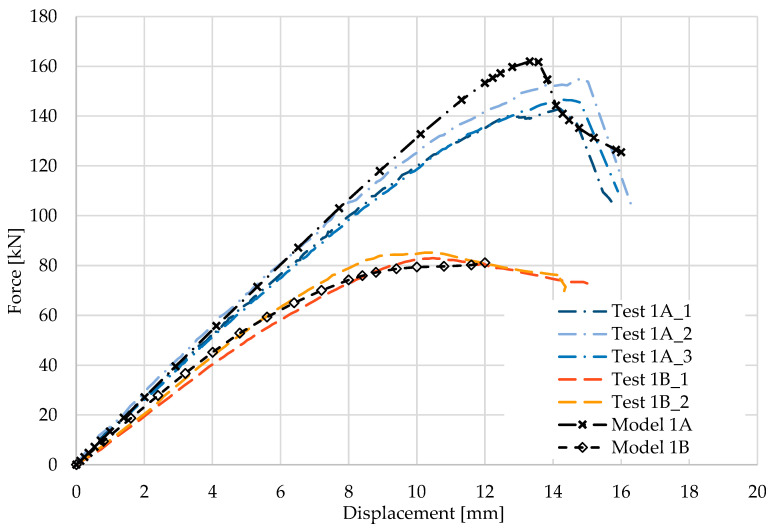
Comparison of force-displacement diagrams from experiment and numerical models.

**Figure 5:**
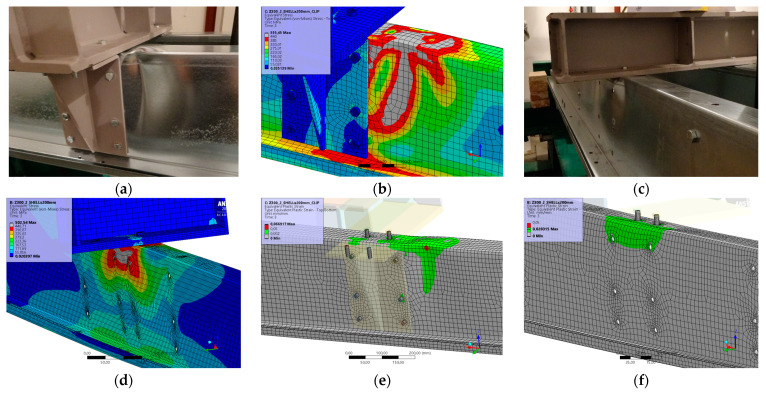
Detail on the support with a clip after reaching the load-bearing capacity (**a**). Von Mises stresses in the range of 0–440 MPa of the support with a clip from the numerical model (**b**). Detail on the support without a clip after reaching the load-bearing capacity (**c**). Von Mises stresses in the range 0–440 MPa on the support without a clip from the numerical model (**d**). Plastic strain in the deformed region of thin-walled purlins after reaching the load-bearing capacity: (**e**) with reinforcing clip, (**f**) without clip.

Figure 4 and Figure 5 show the results from the model configurations with a purlins height of 300 mm; material thickness 1.89 mm; support width 200 mm; and supports span 3.0 m. Values derived from experiments of the same configuration are also shown. The graph includes the variations (A) with a reinforcement clip and (B) without reinforcement.

The force-deformation diagram from the model with the reinforcing clip shows a higher stiffness compared to the experiments and shows a higher load-carrying capacity of about 7%. The numerical model reached a value of 161.2 kN while the experiments reached a value of 149.8 kN. Therefore, the maximum load is also achieved at a lower deformation. This effect can be attributed to an effect of the relatively large imperfections caused by the deformation of the thin-walled beams during erection. These imperfections have not been taken into account in the numerical models. The model without a clip shows a higher initial stiffness compared to the experiment, but the resulting capacity is slightly lower, by about 5% (85.2 kN vs. 80.8 kN). The differences between the models and the experiments may also be due to some extent to the slightly different boundary conditions in the fit of the transom, which was stabilized in the horizontal direction during the experiments by the tested assembly itself.

### 5.2. Result for Model 2A and 2B

**Figure 6:**
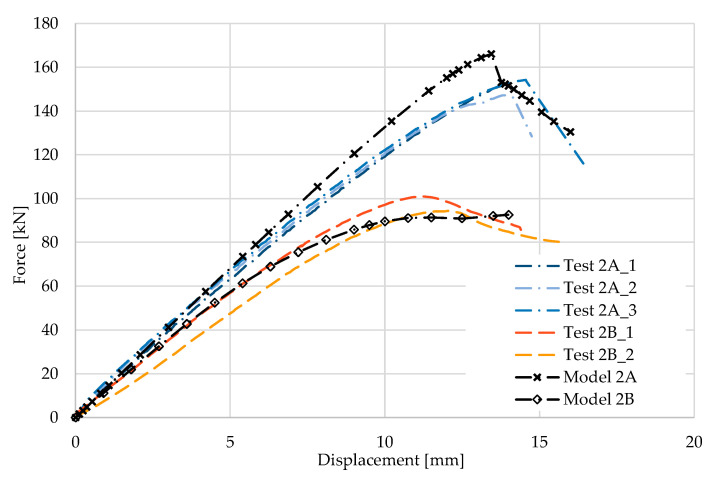
Comparison of force-displacement diagrams from experiment and numerical model.

**Figure 7:**
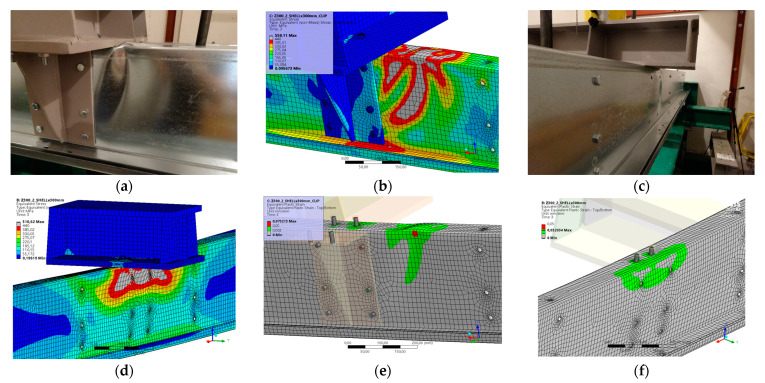
Detail on the support with a clip after reaching the load-bearing capacity (**a**). Von Mises stresses in the range of 0–440 MPa of the support with a clip from the numerical model (**b**). Detail on the support without a clip after reaching the load-bearing capacity (**c**). Von Mises stresses in the range 0–440 MPa on the support without a clip from the numerical model (**d**). Plastic strain in the deformed region of thin-walled purlins after reaching the load-bearing capacity: (**e**) with reinforcing clip, (**f**) without clip.

Figure 6 and Figure 7 show the results from the model configurations with a purlins height of 300 mm; material thickness 1.89 mm; support width 300 mm; and supports span 3.0 m.

Values derived from experiments from the same configuration are also shown. The graph includes the variations (A) with reinforcement clip, and (B) without reinforcement. The force-deformation diagram from the model with the reinforcing clip shows a higher stiffness compared to the experiments and a higher load carrying capacity of about 8% (166.6 kN vs. 152.3 kN). Therefore, the maximum load is also achieved with a lower deformation. The model without a clip shows a higher initial stiffness compared to the experiment, but the resulting capacity is slightly lower, by about 9% (101.1 kN vs. 91.2 kN). The differences between numerical and experimental data are consistent with the 1A and 2A cases.

### 5.3. Result for Model 3A and 3B

**Figure 8:**
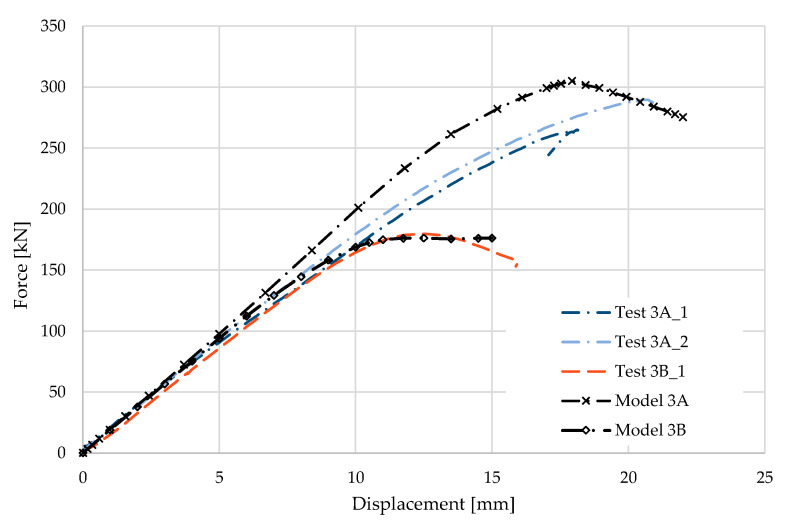
Comparison of force-displacement diagrams from experiment and numerical model.

**Figure 9:**
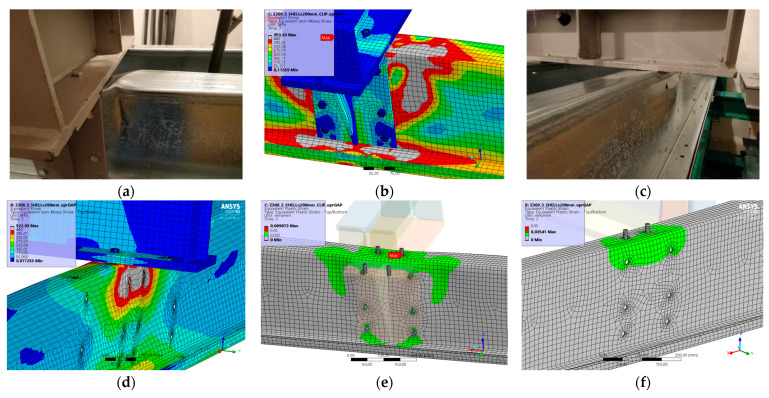
Detail on the support with a clip after reaching the load-bearing capacity (**a**). Von Mises stresses in the range of 0–440 MPa of the support with clip from the numerical model (**b**). Detail on the support without a clip after reaching the load-bearing capacity (**c**). Von Mises stresses in the range 0–440 MPa of the support without a clip from the numerical model (**d**). Plastic strain in the deformed region of thin-walled purlins after reaching the load-bearing capacity: (**e**) with reinforcing clip, (**f**) without clip.

Figure 8 and Figure 9 show the results from the model configurations with a purlins height of 300 mm; material thickness 2.85 mm; support width 200 mm; and supports span 3.0 m. Values derived from experiments from the same configuration are also shown. The graph includes the variations (A) with a reinforcement clip, (B) without reinforcement.

The difference in the stiffnesses results between the numerical models and experiments is about 8% (304.9 kN vs. 280.5 kN). The model without a clip shows a higher initial stiffness compared to the experiment, but the resulting load capacity is slightly higher, about 2% (172.7 kN vs. 176.2 kN). The high level of agreement between the experimental and numerical model results for Model 3B may be due to the higher stiffness of the 2.85-thickness material and the lower influence of stability issues due to the smaller section height and span.

### 5.4. Result for Model 4A and 4B

**Figure 10:**
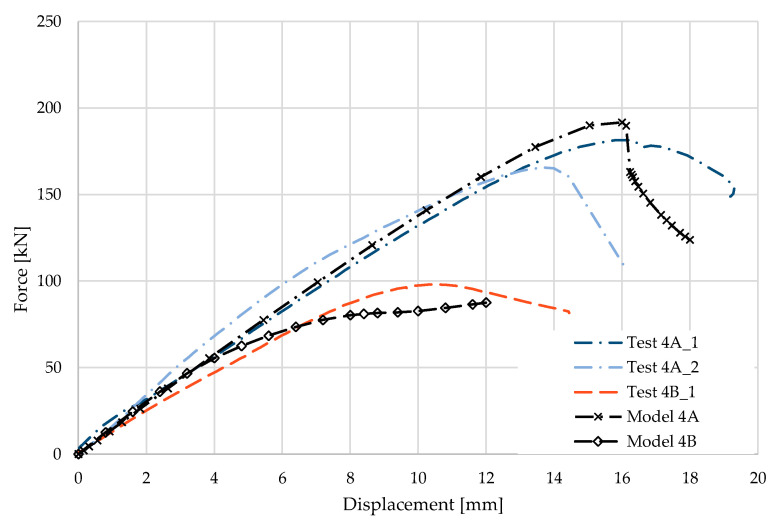
Comparison of force-displacement diagrams from experiment and numerical model.

**Figure 11:**
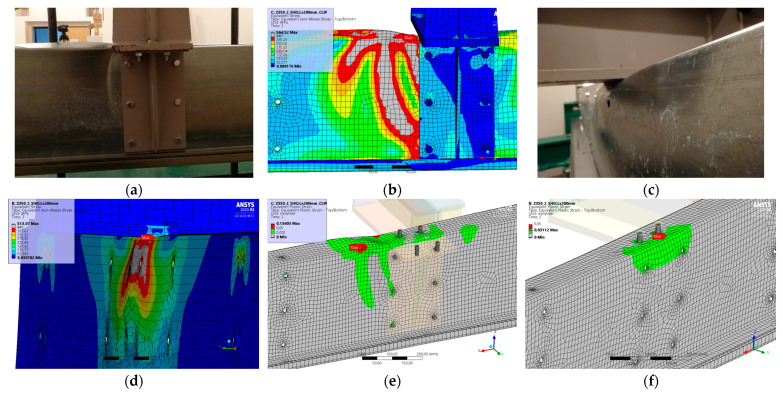
Detail on the support with a clip after reaching the load-bearing capacity (**a**). Von Mises stresses in the range of 0–440 MPa of the support with a clip from the numerical model (**b**). Detail on the support without a clip after reaching the load-bearing capacity (**c**). Von Mises stresses in the range 0–440 MPa of the support without a clip from the numerical model (**d**). Plastic strain in the deformed region of thin-walled purlins after reaching the load-bearing capacity: (**e**) with reinforcing clip, (**f**) without clip.

Figure 10 and Figure 11 show the results from the model configurations with a purlins height of 350 mm; material thickness 1.89 mm; support width 200 mm; and supports span 3.0 m. Values derived from experiments from the same configuration are also shown. The graph includes the variations (A) with a reinforcement clip, (B) without reinforcement.

The force-deformation diagram from the numerical model with the reinforcing clip shows a higher stiffness compared to the experiments and a higher load capacity by about 5% (189.8 kN vs. 180.3 kN). For this reason, the maximum load is also reached at a lower deformation. The reason may be also the neglect of initial imperfections. More is explained in the discussion.

### 5.5. Result for Model 5A and 5B

**Figure 12:**
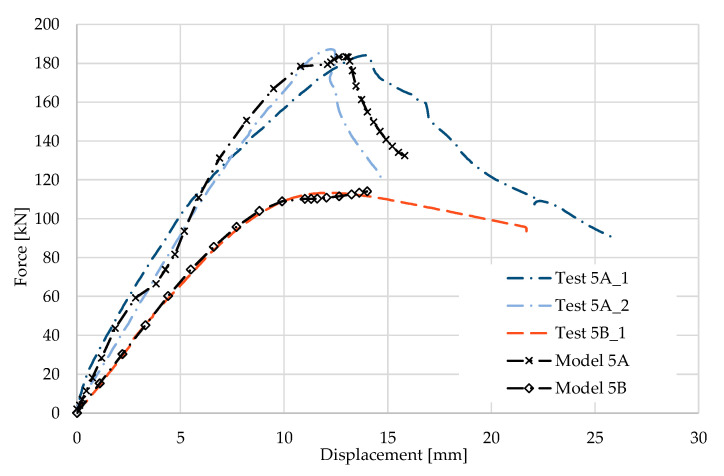
Comparison of force-displacement diagrams from experiment and numerical model.

**Figure 13:**
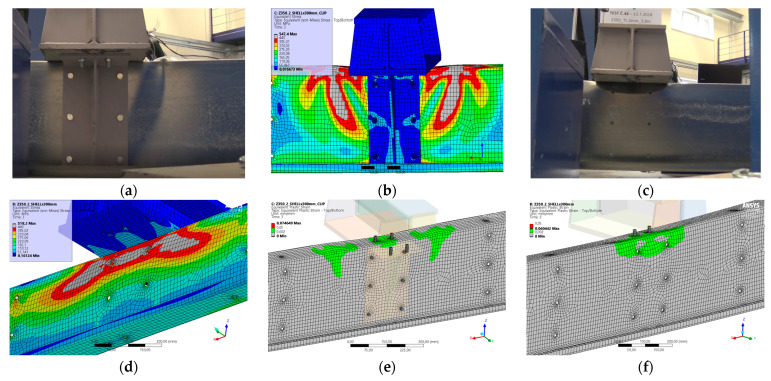
Detail on the support with a clip after reaching the load-bearing capacity (**a**). Von Mises stresses in the range of 0–440 MPa of the support with a clip from the numerical model (**b**). Detail on the support without a clip after reaching the load-bearing capacity (**c**). Von Mises stresses in the range 0–440 MPa of the support without a clip from the numerical model (**d**). Plastic strain in the deformed region of thin-walled purlins after reaching the load-bearing capacity: (**e**) with reinforcing clip, (**f**) without clip.

Figure 12 and Figure 13 show the results from the model configurations with a purlins height of 350 mm; material thickness 1.89 mm; support width 300 mm; and supports span 3.0 m.

Values derived from experiments from the same configuration are also shown. The graph includes the variations (A) with a reinforcement clip, (B) without reinforcement. The force-deformation diagram of the model with a reinforcing clip shows slightly higher stiffness compared to the experiments. The differences in the load capacities are only about 2% (183.2 kN vs. 179.5 kN). The maximum load in the model occurs at almost identical displacement of the transom. The result from the model without a clip is almost identical to the experiment. There are minor differences in the shape of the failure mode, which comes out more symmetrically in the models than in the corresponding experiments. This is an expected behaviour, since the imperfections arising in the model are symmetrical in front of and behind the transom compared to the real beams.

### 5.6. Result for Model 6A and 6B

**Figure 14:**
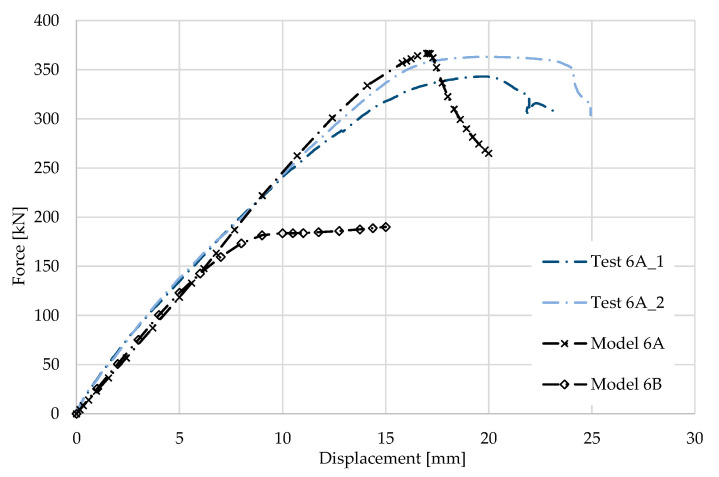
Comparison of force-displacement diagrams from experiment and numerical model. The experiment without a clip was not performed, so the results are not presented.

**Figure 15:**
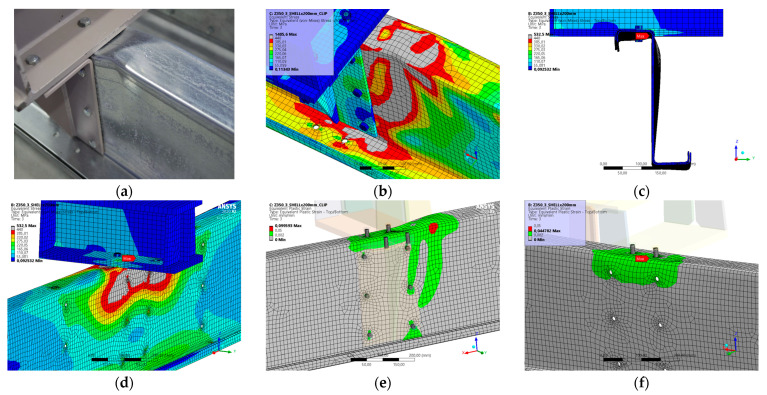
Detail on the support with a clip after reaching the load-bearing capacity (**a**). Von Mises stresses in the range of 0–440 MPa of the support with a clip from the numerical model (**b**). Model of the cross section of the support without a clip (**c**). Von Mises stresses in the range 0–440 MPa of the support without a clip from the numerical model (**d**). Plastic strain in the deformed region of thin-walled purlins after reaching the load-bearing capacity: (**e**) with reinforcing clip, (**f**) without clip.

Figure 14 and Figure 15 show the results from the model configurations with a purlins height of 350 mm; material thickness 2.85 mm; support width 200 mm; and supports span 3.0 m. Values derived from experiments from the same configuration are also shown. The graph includes the variations (A) with a reinforcement clip, (B) without reinforcement.

The force-deformation diagram for the model with the reinforcing clip shows a higher stiffness compared to the experiments and also a higher load capacity of about 1% (366.2 kN vs. 362.8 kN). The detail of the connection without the clip was not verified experimentally. However, a numerical model has been developed. The differences between the models and experiments are not very large in terms of global behaviour.

### 5.7. Result for Model 7A and 7B

**Figure 16:**
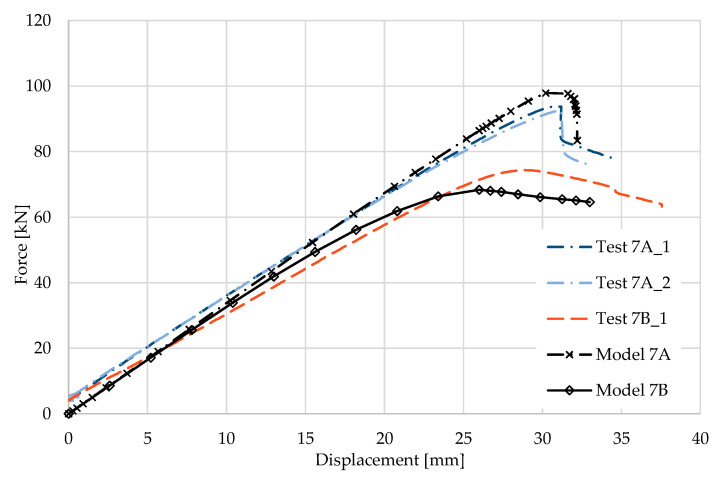
Comparison of force-displacement diagrams from experiment and numerical model.

**Figure 17:**
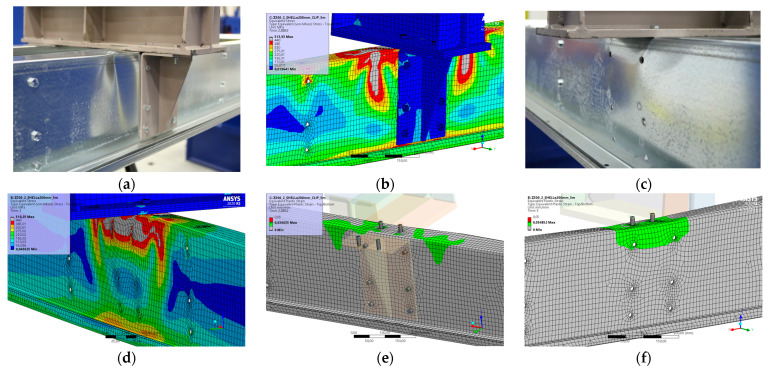
Detail on the support with a clip after reaching the load-bearing capacity (**a**). Von Mises stresses in the range of 0–440 MPa of the support with a clip from the numerical model (**b**). Detail on the support without a clip after reaching the load-bearing capacity (**c**). Von Mises stresses in the range 0–440 MPa of the support without a clip from the numerical model (**d**). Plastic strain in the deformed region of thin-walled purlins after reaching the load-bearing capacity: (**e**) with reinforcing clip, (**f**) without clip.

Figure 16 and Figure 17 show the results from the model configurations with a purlins height of 300 mm; material thickness 1.89 mm; support width 200 mm; and supports span 5.1 m. Values derived from experiments from the same configuration are also shown. The graph includes the variations (A) with a reinforcement clip, (B) without reinforcement.

Comparison of the results for the layout with the reinforcing clip shows a slightly higher stiffness. The difference in load capacity is only about 4% (98.9 kN vs. 94.9 kN). The maximum load is achieved in the model with almost identical displacement of the partition. The differences between the models and experiments are minimal in terms of the global behaviour represented by the load-deformation diagrams. There are minor differences in the load capacity values, especially for the fits without bracing.

### 5.8. Result for Model 8A and 8B

**Figure 18:**
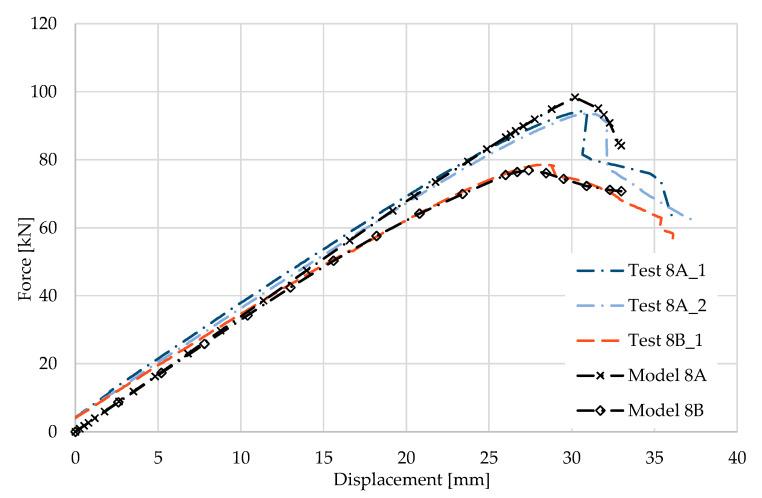
Comparison of force-displacement diagrams from experiment and numerical model.

**Figure 19:**
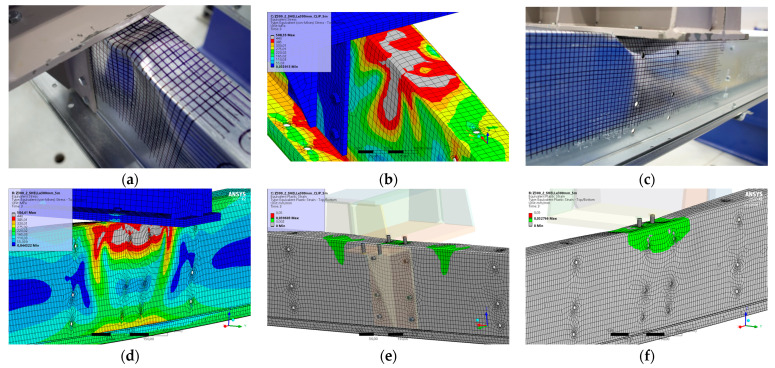
Detail on the support with a clip after reaching the load-bearing capacity (**a**). Von Mises stresses in the range of 0–440 MPa of the support with a clip from the numerical model (**b**). Detail on the support without a clip after reaching the load-bearing capacity (**c**). Von Mises stresses in the range 0–440 MPa of the support without a clip from the numerical model (**d**). Plastic strain in the deformed region of thin-walled purlins after reaching the load-bearing capacity: (**e**) with reinforcing clip, (**f**) without clip.

Figure 18 and Figure 19 show the results from the model configurations with a purlins height of 300 mm; material thickness 1.89 mm; support width 300 mm; and supports span 5.1 m. Values derived from experiments from the same configuration are also shown. The graph includes the variations (A) with a reinforcement clip, (B) without reinforcement.

The results from the model with a reinforcement clip show slightly higher stiffness compared to the experiments. The difference in the load capacity is only about 4% (98.4 kN vs. 94.4 kN). The maximum load is reached in the model with almost identical displacement of the transom as it was in the experiment. The model without the clip shows a similar initial stiffness to the experiment. The resulting load capacity is slightly lower by about 2% (76.1 kN vs. 74.6 kN). The differences between the models and experiments are thus minimal.

### 5.9. Result for Model 9A and 9B

**Figure 20:**
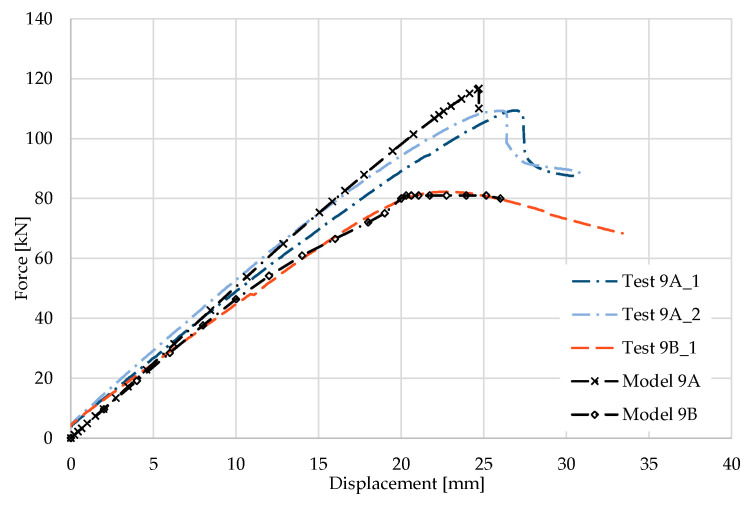
Comparison of force-displacement diagrams from experiment and numerical model.

**Figure 21:**
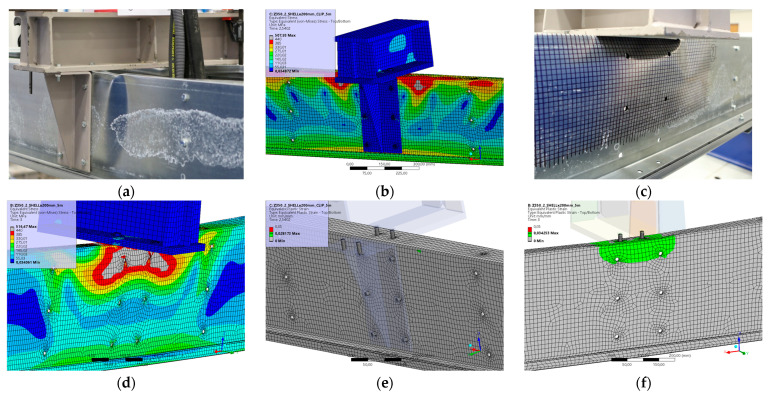
Detail on the support with a clip after reaching the load-bearing capacity (**a**). Von Mises stresses in the range of 0–440 MPa of the support with a clip from the numerical model (**b**). Detail on the support without a clip after reaching the load-bearing capacity (**c**). Von Mises stresses in the range 0–440 MPa of the support without a clip from the numerical model (**d**). Plastic strain in the deformed region of thin-walled purlins after reaching the load-bearing capacity: (**e**) with reinforcing clip, (**f**) without clip.

Figure 20 and Figure 21 show the results from the model configurations with a purlins height of 350 mm; material thickness 1.89 mm; support width 200 mm; and supports span 5.1 m. Values derived from experiments from the same configuration are also shown. The graph includes the variations (A) with a reinforcement clip, (B) without reinforcement.

Consistently with several previous configurations, it can be observed that the force-deformation diagram from the numerical model with the reinforcing clip shows a slightly higher stiffness compared to the experiments. The differences in the load capacities are about 6% (116.3 kN vs. 109.3 kN). The model without a clip shows almost the same behaviour.

### 5.10. Result for Model 10A and 10B

**Figure 22:**
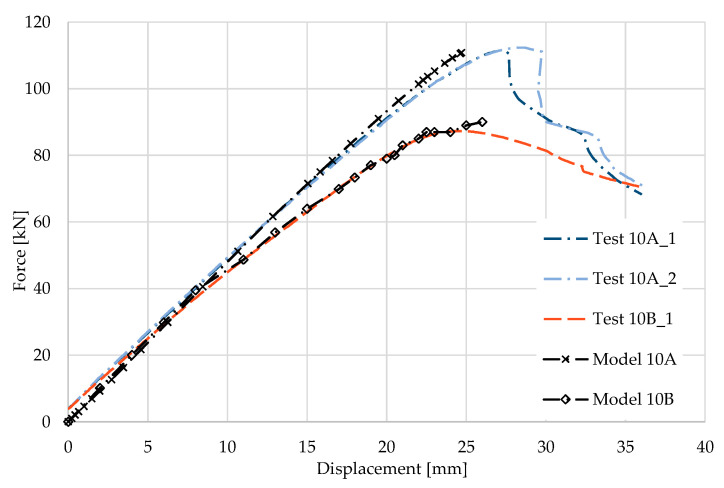
Comparison of force-displacement diagrams from experiment and numerical model.

**Figure 23:**
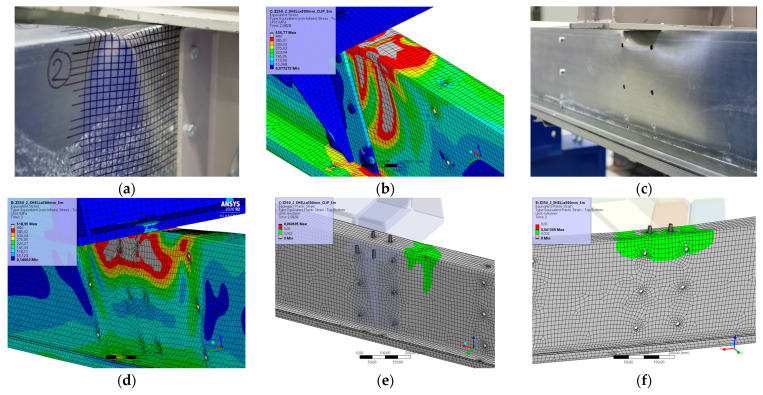
Detail on the support with a clip after reaching the load-bearing capacity (**a**). Von Mises stresses in the range of 0–440 MPa of the support with a clip from the numerical model (**b**). Detail on the support without a clip after reaching the load-bearing capacity (**c**). Von Mises stresses in the range 0–440 MPa of the support without a clip from the numerical model (**d**). Plastic strain in the deformed region of thin-walled purlins after reaching the load-bearing capacity: (**e**) with reinforcing clip, (**f**) without clip.

Figure 22 and Figure 23 show the results from the model configurations with a purlins height of 350 mm; material thickness 1.89 mm; support width 300 mm; and supports span 5.1 m. Values derived from experiments from the same configuration are also shown. The graph includes the variations (A) with a reinforcement clip, (B) without reinforcement.

The difference in load capacity is about 3% (110.8 kN vs. 107.5 kN). The maximum load is reached in the model with almost identical displacement of the transom. Minor differences are due to imperfections in the thin-walled beams. These imperfections have not been taken into account in the numerical models. The model without a clip shows an initial higher stiffness compared to the experiment, but the resulting capacity is about 1% lower (85.2 kN vs. 84.4 kN).

## 6. Discussion

Measured data from physical experiments [24] were used to validate the numerical models. These numerical models can be used to realistically simulate the detailed behaviour of the connections of the TW-CFS purlins to the main structure and their further modifications. The comparison shows that the numerical models exhibit higher stiffness compared to the experiments, mostly under higher loads. This is attributed to the possible influence of imperfections and other design and installation tolerances; for example, in the misalignment of the bolt holes and the resulting differential settlement of the individual parts of the tested assembly during loading. This is consistent with the findings of other similar investigations [20,29].

In terms of load carrying capacity, the numerical models of the connections with the clip were more load carrying than the experiments, ranging from 1 to 8%. The differences for the connections without the reinforcing clip were higher, ranging from 2 to 13%. The best agreement between the numerical model and the experiment was obtained for model 07 and model 08. Minor differences are in the shape of the mode of failure, which comes out more symmetrically in the models compared to the experiments. This is an expected effect, since the imperfections arising in the model are symmetrical in front of and behind the transom compared to the real beams. In general, the models and experiments agreed better with the fit with a clip than without a clip. It is necessary to add a remark to all results that the numerical models did not take into account imperfections that can be introduced into the model, but this would bring additional problems. The result shows that even without taking imperfections into account, the resulting load capacities are similar to the numerical models with respect to the accuracy of the measurements achieved. For example, for the clipless details, the load capacities in the models were lower than in the experiment, indicating a beneficial effect of imperfections. On the other hand, it was rather the opposite for the details with a clip. Here, too, the introduction of imperfections during assembly may have played a role–not all holes interfaced, and so the beams were deformed differently when the bolts were tightened. Again, this would have been a problem to introduce into the model, as the hole inaccuracies were also largely random across the beam portfolio.

Numerically, the final collapse observed in the experiment was not always achieved. The numerical simulation was terminated when the maximum load was reached with an overlap in the downward part of the working diagram, or when the maximum force was reached without further development of deformation after crippling of the thin-walled truss sections. The phase corresponding to the end of the numerical calculation was not documented in photographic detail during the experiment.

This is one of the reasons why not all deformation shapes resulting from the numerical simulations correspond to the compared photographs. Another reason could be the influence of various initial imperfections that affected the initialization and subsequent progression of the collapsed shape of the TW-CFS purlins [30].

## 7. Conclusions

This article presented a way to use accurate numerical models of TW-CFS high purlins used in construction. Models were validated using results from experiments. The designs of 300 mm and 350 mm high Z cross-section purlins were presented in several variations of span and support width, material thickness and connection type.

It was found that the chosen procedure for the development of the numerical models gives results close enough to the experiments performed (expressed differences for the fits with and without clip up to 8% and 11% in the maximum load transferred), and it is therefore possible to use this procedure to complement the experimental campaigns. The possibility of using FEM modelling of the studied roof truss connection details was verified. The load capacity results obtained by numerical simulations were in good agreement with the experimental measurements for the experimentally verified materials. The sophisticated numerical models could be used for possible interpolation of intermediate values that were not directly part of the test campaign based on their accuracy and agreement with the experimental measurements. In this way, numerical models of other engagement variants could be constructed, and their results could be used to predict the actual shape of the interacting M/R (moment/reaction) capacity diagram. Further research is expected to focus on specific truss types and material classes. A shift in the accuracy of the numerical model results can be expected by introducing imperfections, but this is more difficult mainly due to the different behaviours of different combinations of sample shapes, lengths and thicknesses.

## Figures and Tables

**Table 1:** Materials parameters of numerical models.

Young’s Modulus *E* [MPa]	Poisson’s Ratio *ν* [-]	Yield Strength *f*y [MPa]
200,000	0.3	440

**Table 2:** Labelling of numerical models with studied variable input parameters.

Model Number	Span of Supports	Z Cross-Section Heights	Support Connection	Thickness of Material	Support Width	Graph Labelling
1A	3.0 m	300 mm	with clip	1.89	200 mm	Z300_1.89_200_3.0_A
1B	3.0 m	300 mm	without clip	1.89	200 mm	Z300_1.89_200_3.0_B
2A	3.0 m	300 mm	with clip	1.89	300 mm	Z300_1.89_300_3.0_A
2B	3.0 m	300 mm	without clip	1.89	300 mm	Z300_1.89_300_3.0_B
3A	3.0 m	300 mm	with clip	2.85	200 mm	Z300_2.85_200_3.0_A
3B	3.0 m	300 mm	without clip	2.85	200 mm	Z300_2.85_200_3.0_B
4A	3.0 m	350 mm	with clip	1.89	200 mm	Z350_1.89_200_3.0_A
4B	3.0 m	350 mm	without clip	1.89	200 mm	Z350_1.89_200_3.0_B
5A	3.0 m	350 mm	with clip	1.89	300 mm	Z350_1.89_300_3.0_A
5B	3.0 m	350 mm	without clip	1.89	300 mm	Z350_1.89_200_3.0_B
6A	3.0 m	350 mm	with clip	2.85	200 mm	Z350_2.85_200_3.0_A
6B	3.0 m	350 mm	without clip	2.85	200 mm	Z350_2.85_200_3.0_B
7A	5.1 m	300 mm	with clip	1.89	200 mm	Z300_1.89_200_5.1_A
7B	5.1 m	300 mm	without clip	1.89	200 mm	Z300_1.89_200_5.1_B
8A	5.1 m	300 mm	with clip	1.89	300 mm	Z300_1.89_300_5.1_A
8B	5.1 m	300 mm	without clip	1.89	300 mm	Z300_1.89_300_5.1_B
9A	5.1 m	350 mm	with clip	1.89	200 mm	Z350_1.89_200_5.1_A
9B	5.1 m	350 mm	without clip	1.89	200 mm	Z350_1.89_200_5.1_B
10A	5.1 m	350 mm	with clip	1.89	300 mm	Z350_1.89_300_5.1_A
10B	5.1 m	350 mm	without clip	1.89	300 mm	Z350_1.89_300_5.1_B

## Data Availability

Data are contained within the article.
